# Angle-Insensitive Defect-Mode Absorption in Photonic Crystals Containing Hyperbolic Metamaterials

**DOI:** 10.3390/nano16160985

**Published:** 2026-08-10

**Authors:** Mingyang Liu, Guang Lu, Bing Wang

**Affiliations:** 1 School of Space Science and Physics, Shandong University, Weihai 264209, China; 202538111@mail.sdu.edu.cn (M.L.); wbing@sdu.edu.cn (B.W.); 2 Laboratory for Electrom Agnetic Detection (LEAD), Institute of Space Sciences, Shandong University, Weihai 264209, China

**Keywords:** hyperbolic metamaterials, photonic crystals, defect mode, angle-insensitive, optical absorber

## Abstract

Omnidirectional optical devices are essential for photodetection, thermal radiation regulation, and solar energy harvesting. However, the photonic bandgaps and defect modes of conventional one-dimensional photonic crystals (1DPCs) are constrained by the Bragg scattering condition, leading to strong angular dependence that substantially limits their practical applications over wide angle ranges. In this work, we theoretically design and experimentally verify an angle-insensitive photonic crystal defect-mode absorber based on hyperbolic metamaterials (HMMs). Leveraging the unique isofrequency dispersion of HMMs, we introduce a phase compensation mechanism into a photonic crystal composed of alternating HMM and dielectric layers. Calculations show that inserting a metallic defect layer excites a highly localized defect mode within the bandgap, whose resonant wavelength remains almost unchanged with incident angle. To simplify fabrication and enhance absorption, we reduce the number of periods and design a heterostructure containing subwavelength Ag/TiO_2_ multilayers. Measurements under TM polarization over 0–70° show that the defect-mode peak shifts by only 3.5 nm, while the absorptance decreases from ~0.717 at normal incidence to ~0.292 at 70°. This study provides an effective strategy for designing and fabricating resonance wavelength angle-insensitive optical absorbers enabled by HMM-based phase compensation.

## 1. Introduction

Omnidirectional optical devices—such as perfect absorbers and omnidirectional reflectors—are essential for modern optical systems, solar energy harvesting, photodetection, and thermal radiation management [1,2]. Nevertheless, most optical absorbers exhibit strong angular dependence, and achieving stable optical responses over a wide range of incident angles remains a key challenge [3,4,5].

One-dimensional photonic crystals (1DPCs) have attracted extensive interest owing to their photonic bandgaps (PBGs), which tailor electromagnetic wave propagation [6,7]. Introducing a defect layer into the periodic lattice excites strongly localized defect modes within the PBG, whereas Tamm plasmon polaritons (TPPs) emerge at metal–PC interfaces [8,9]. However, conventional all-dielectric 1DPCs are subject to an intrinsic constraint: their PBGs and defect modes are highly angle-sensitive owing to the Bragg condition. Consequently, both the bandgaps and the defect modes blueshift with increasing incident angle [10,11].

Hyperbolic metamaterials (HMMs) have therefore been exploited to overcome this angular limitation in optical device design [12,13,14]. As anisotropic metamaterials, HMMs exhibit hyperbolic isofrequency curves under transverse magnetic (TM) polarization [15,16]. Inside HMMs, the longitudinal wavevector component and the propagation phase both increase monotonically with the transverse wavevector, which is determined by the incident angle. Owing to the opposite phase evolution of HMMs and conventional dielectrics, optical heterostructures with angle-insensitive or even redshifted bandgaps can be realized via the phase compensation effect [17,18,19]. Recent studies further show that HMM-based structures enable wide-angle perfect absorption, robust topological interface states, and Fano resonances [20,21,22].

Previous studies have verified that metal-dielectric multilayers can act as hyperbolic metamaterials (HMMs) to achieve angle-insensitive photonic bandgaps [23], perfect optical absorbers [24], and redshift-tunable bandgaps via phase-matching engineering [25]. These studies have laid a solid theoretical basis for HMM-enabled angle-insensitive structural designs. Nevertheless, few efforts have investigated localized defect-mode absorption within such bandgaps. In this work, we embed an ultrathin metallic defect layer into the HMM-integrated angle-insensitive photonic crystal. The introduced defect layer excites a strongly localized defect mode inside the stop band, enabling near-unity light absorption that maintains robust angular insensitivity. Distinct from conventional HMM-based absorbers dependent on band-edge or Tamm-state resonances, our architecture realizes angle-immune defect-mode absorption accompanied by extreme electromagnetic field confinement. To our knowledge, this work presents the first experimental demonstration of such defect-mode absorbers in HMM-incorporated photonic crystals. The target structure is fabricated via ion-assisted electron-beam evaporation, and its optical performance is characterized using angle-resolved spectrophotometry; corresponding measured results are discussed in subsequent sections.

## 2. Structure Design and Theoretical Model

**Figure 1:**
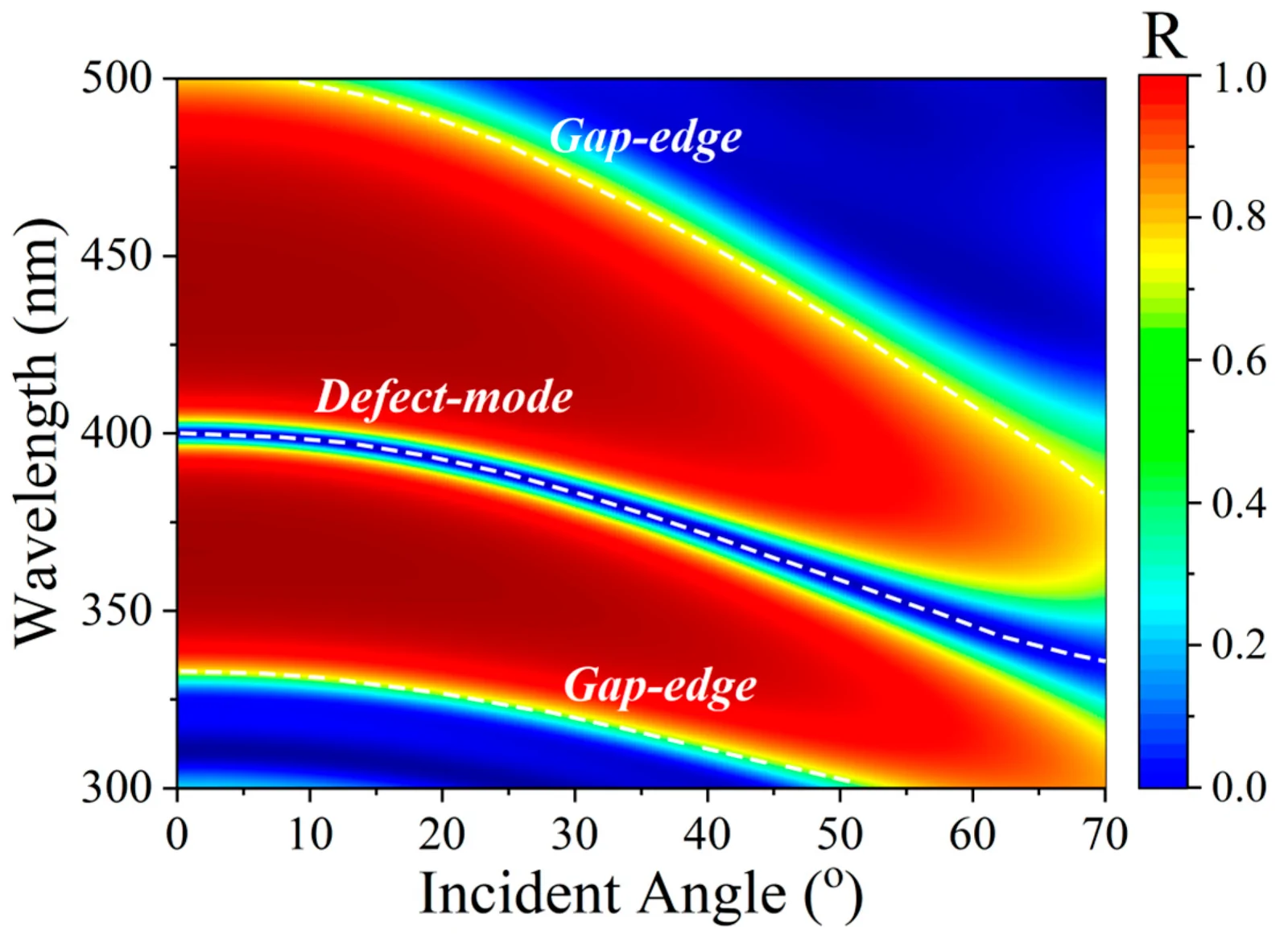
Simulated reflection spectra of the defect mode in a conventional all-dielectric one-dimensional photonic crystal.

As a reference, we first investigate the angular dispersion of the defect mode in a conventional all-dielectric photonic heterostructure (AB)^3^C(AB)^4^. The structure consists of low- and high-index layers A (SiO_2_, *n_A_* = 1.43, *d_A_* = 69.9 nm) and B (TiO_2_, *n_B_* = 2.12, *d_B_* = 47.1 nm), where the refractive indices are fitted from experimental data [23,24,25]. The inserted defect layer C is a 25 nm thick TiO_2_ film. Figure 1 presents the transverse magnetic (TM) reflection spectra simulated via the transfer matrix method (TMM). At normal incidence (0°), a distinct reflection dip induced by the strongly localized defect mode is clearly observed at 400 nm within the photonic bandgap (PBG). However, as the incident angle increases from 0° to 70°, both the defect-mode dip and the PBG edges exhibit a pronounced blueshift. This intrinsic angular dispersion characteristic stems from the in-plane wavevector matching of isotropic dielectrics, which strictly drives the resonance toward higher frequencies under oblique incidence to satisfy the Bragg condition.

**Figure 2:**
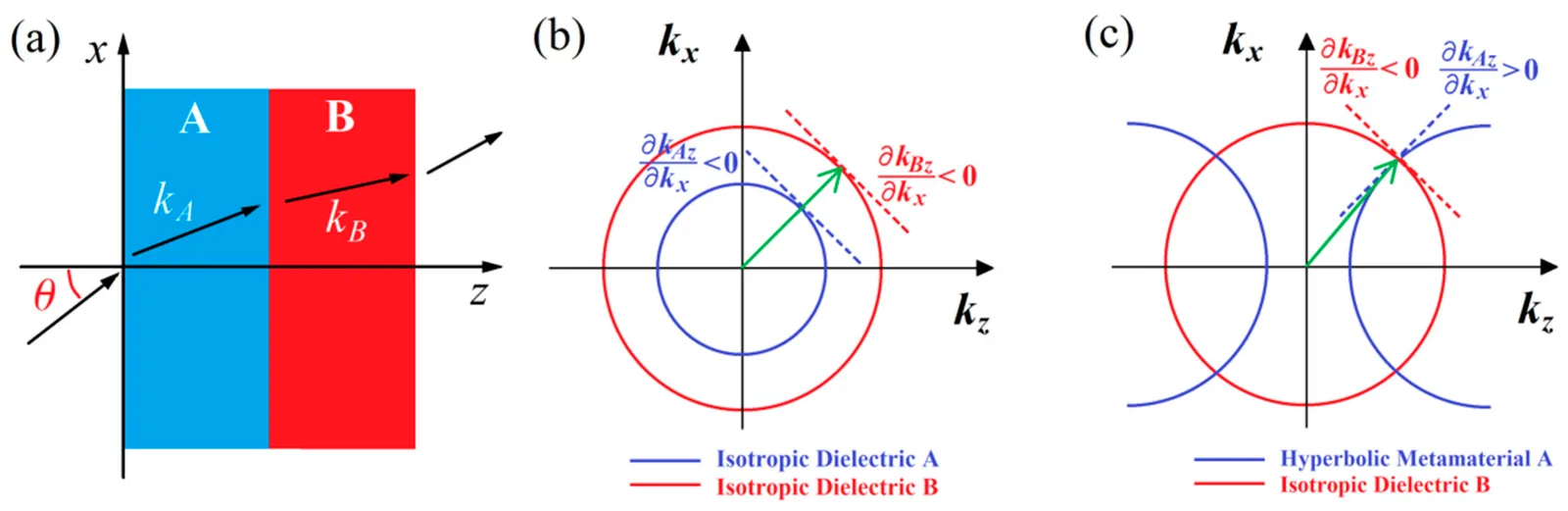
(**a**) Schematic of the unit cell. Isofrequency curves of the constituent media in (**b**) the conventional all-dielectric 1DPC and (**c**) the 1DPC containing HMMs and dielectrics.

For a conventional all-dielectric one-dimensional photonic crystal (1DPC) (AB)^N^, layers A and B feature circular isofrequency curves, as illustrated in Figure 2a. The transverse wavevector *k_x_* rises with the incident angle, which simultaneously reduces the longitudinal wavevectors *k_Az_* and *k_Bz_* inside both dielectric layers. The photonic bandgap (PBG) of 1DPCs is dominated by the Bragg scattering condition. As the incident angle grows, resonant frequencies shift to higher values to satisfy the Bragg criterion, yielding a blueshifted PBG.

In sharp contrast, Figure 2b shows that hyperbolic metamaterials (HMMs) support open hyperbolic dispersion under specific polarizations. For HMM films, rising *k_x_* enlarges the longitudinal wavevector *k_Az_*, as well as its associated propagation phase. By contrast, *k_Bz_* in conventional dielectrics decreases with growing *k_x_* This leads to opposite phase evolution in the two material types, i.e., their phase derivatives with respect to *k_x_* carry opposite signs.

Accordingly, alternating HMM and dielectric layers forms a photonic heterostructure that leverages the phase variation compensation effect to realize angle-insensitive PBGs (zero-shift bandgaps). Moreover, inserting a defect layer to break the lattice periodicity of this angularly robust photonic crystal excites localized defect modes that remain insensitive to incident angles within the bandgap.

Hyperbolic metamaterials (HMMs) represent a class of highly anisotropic metamaterials. Here, we consider electrically tunable two-dimensional (2D) HMMs. Under transverse magnetic (TM) polarization, the isofrequency curve of a 2D HMM can be described as:

(1) $$\frac{k_{x}^{2}}{\varepsilon_{Az}}+\frac{k_{Az}^{2}}{\varepsilon_{Ax}}=k_{0}^{2}$$

Conversely, under transverse electric (TE) polarization, the isofrequency curve is governed by $k_{x}^{2}+k_{AZ}^{2}=\varepsilon_{Ax}k_{0}^{2}$. Consequently, the electrically tunable HMM exhibits hyperbolic dispersion characteristics exclusively under TM polarization; thus, our discussion is restricted solely to the TM polarization case. In HMMs, the real parts of the diagonal permittivity components, *ε_Ax_* and *ε_Az_*, possess opposite signs. Specifically, the medium is classified as a Type-I HMM when *ε_Ax_* > 0 and *ε_Az_* < 0, whereas it behaves as a Type-II HMM when *ε_Ax_* < 0 and *ε_Az_* > 0.

Metallic silver (Ag) exhibits negative permittivity at frequencies below its plasma frequency. The dispersion of Ag thin films is described by the Lorentz–Drude model [23,24,25], expressed as:

(2) $$\varepsilon_{M}=\left[1-f_{0}\frac{\omega_{p}^{2}}{\omega_{2}+i\gamma_{0}\omega }\right]-f_{1}\frac{\omega_{p}^{2}}{\omega_{2}-\omega_{1}^{2}+i\gamma_{1}\omega }$$

where *f*_0_ = 0.8213, *ω_p_* = 1.2983 × 1015 Hz, *γ*_0_ = 1.2983 × 1015 Hz, *f*_1_ = 0.8213, *ω*_1_ = 0.6630*ω_p_*, and *γ*_1_ = 0.9044 × 1015 Hz denote the fitting parameters, all extracted from our experimental characterizations of standalone Ag films.

In contrast, dielectric TiO_2_ maintains nearly constant positive permittivity across the visible optical band. Based on effective medium theory (EMT), subwavelength multilayers with alternating Ag (M) and TiO_2_(D) layers can be homogenized as anisotropic bulk media. Owing to the layered stacking geometry, the effective permittivities along the *x* and *z* axes differ substantially. The principal elements of the effective permittivity tensor for this metal-dielectric multilayer are derived as follows:

(3) $$\varepsilon_{Ax}=\varepsilon_{D}(1-p)+\varepsilon_{M}p$$

(4) $$\varepsilon_{Az}=\frac{1}{\frac{1-p}{\varepsilon_{D}}+\frac{p}{\varepsilon_{M}}}$$

where *p* stands for the metal filling ratio.

It should be noted that the EMT is applicable when the multilayer period is much smaller than the wavelength of interest, and each constituent layer is sufficiently thin compared to the wavelength.

**Figure 3:**
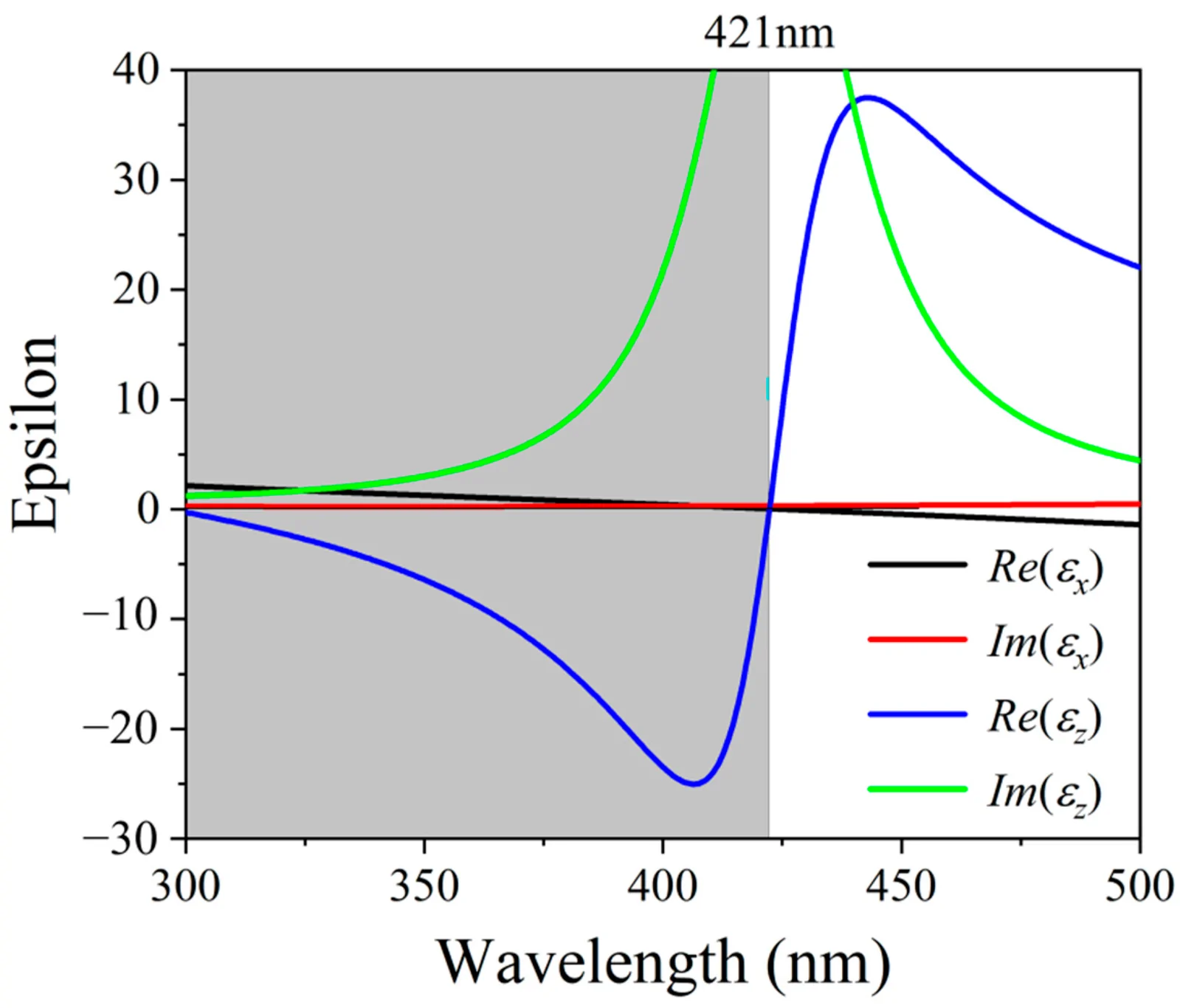
Effective permittivity spectra of the subwavelength metal-dielectric multilayer composed of Ag and TiO_2_ with a filling ratio of *p* = 0.5.

We adopt a representative geometry with *p* = 0.5. Figure 3 presents the calculated effective permittivity spectra of the Ag/TiO_2_ multilayer as a function of wavelength. The shaded region marks the spectral range supporting hyperbolic dispersion. Within this range, the relations *ε_Ax_* > 0 and *ε_Az_* < 0 hold, confirming that the multilayer operates as a Type-I HMM.

**Figure 4:**
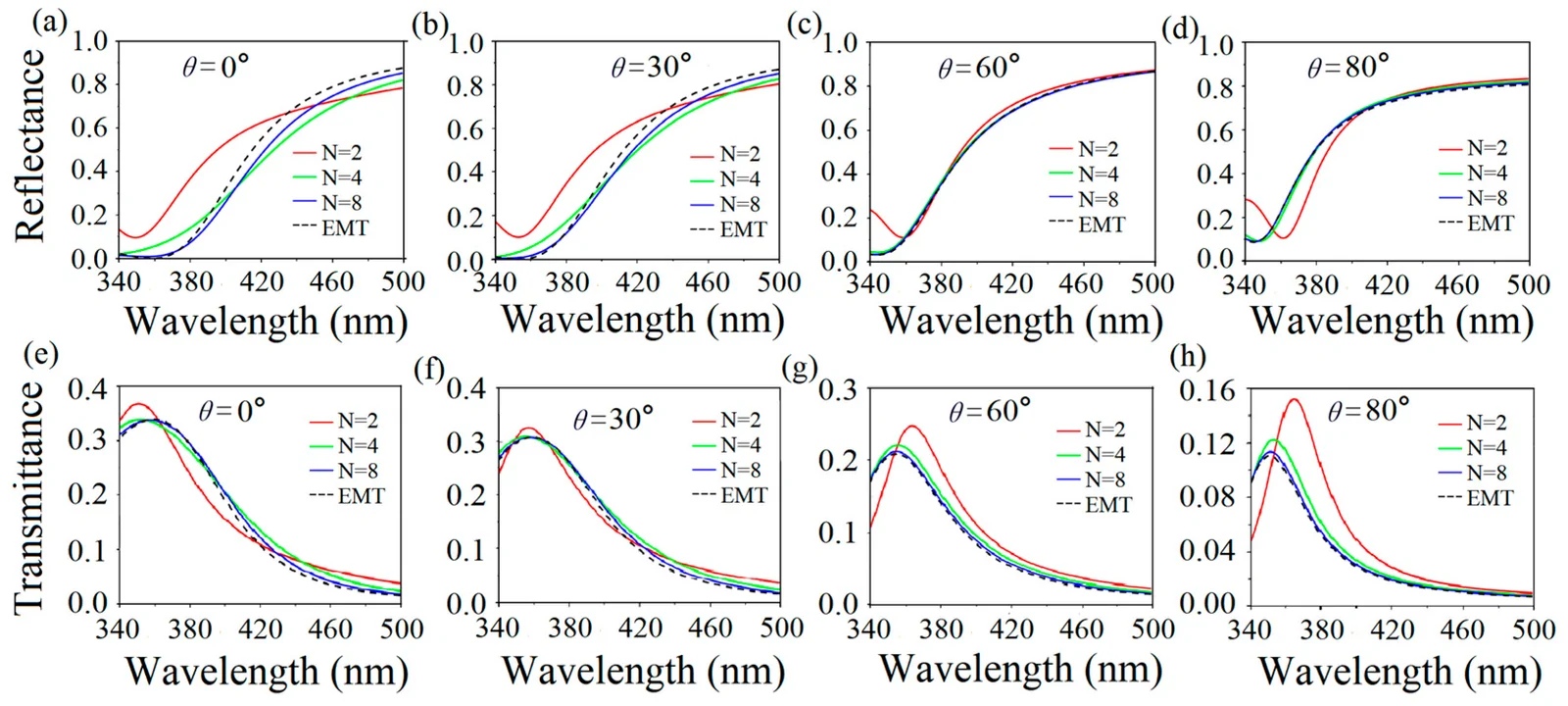
Calculated TM-polarized (**a**–**d**) reflection and (**e**–**h**) transmission spectra of the (AB)^N^ multilayer with N = 2, 4, 8 (red, green, and blue solid lines) at incident angles of 0°, 30°, 60°, and 80° for reflection (**a**–**d**) and 0°, 30°, 60°, and 80° for transmission (**e**–**h**). The black dashed curves correspond to the EMT-based homogeneous layer.

Before applying the effective medium approximation to describe the HMM layer, we first verify its validity for our specific structure with only two Ag/TiO_2_ periods. Figure 4 compares the reflection and transmission spectra of the actual (AB)^N^ multilayer (N = 2, 4, 8) with those of the effective homogeneous layer derived from EMT, calculated at incident angles of 0°, 30°, 60°, and 80° under TM polarization. For N = 4 and 8, the multilayer spectra are nearly identical to the homogeneous-layer results, and the agreement improves with increasing N, as expected. Notably, even for N = 2, the spectra show good agreement with the EMT predictions, with only minor deviations. This confirms that the two-period (AB)^2^ multilayer can be reliably treated as an effective uniform HMM layer in our calculations. Although larger N would improve the EMA accuracy, we choose N = 2 as a practical trade-off to avoid the increased fabrication complexity and additional losses associated with a larger number of ultrathin Ag layers.

**Figure 5:**
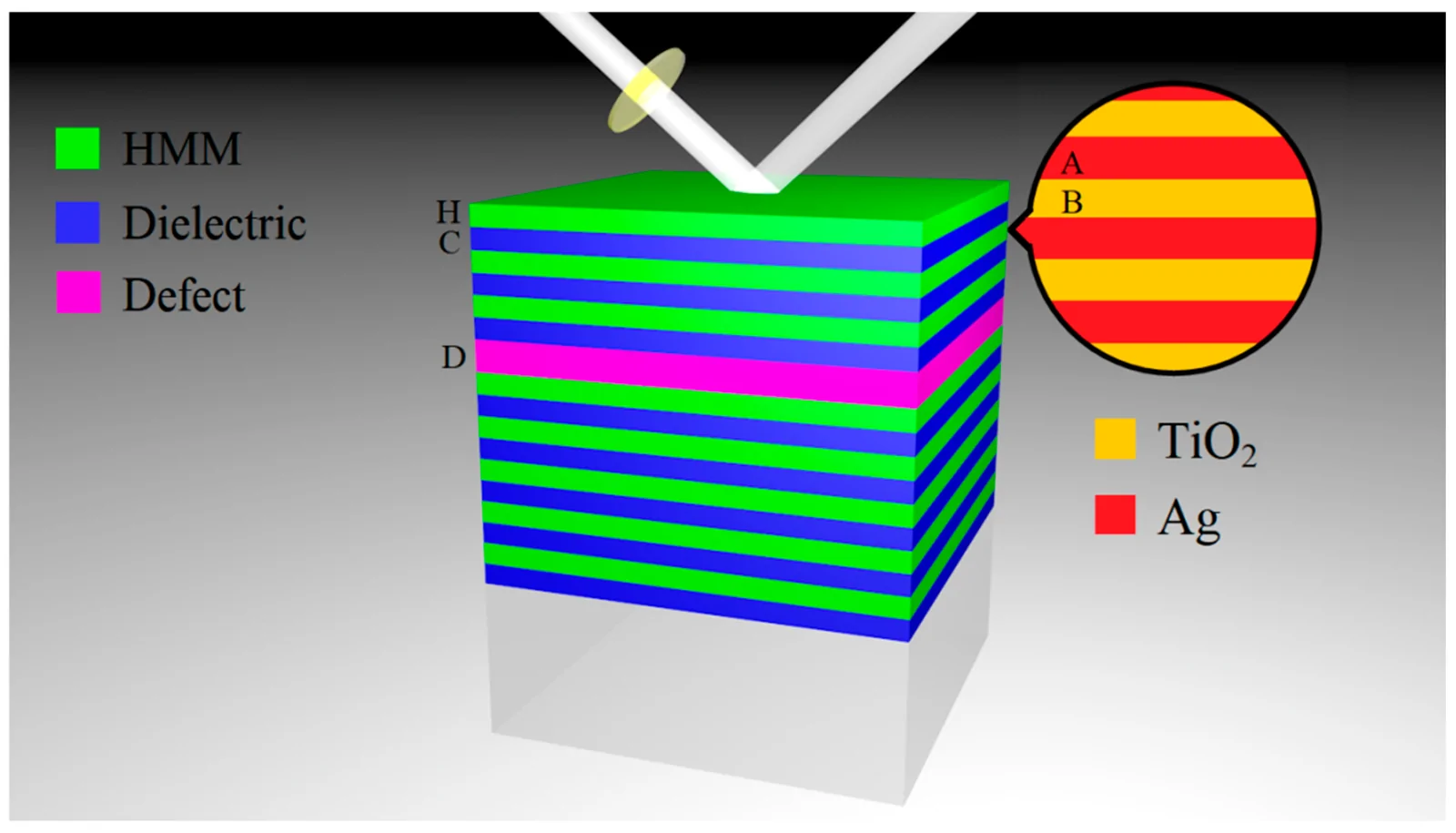
Schematic illustration of the photonic crystal heterostructure containing hyperbolic metamaterials with a central defect layer. The H layer corresponds to the HMM constructed by periodic alternation of TiO_2_ layers (A) and silver layers (B); Layer C refers to the dielectric medium layer, whilst Layer D acts as the central defect layer.

Based on the phase variation compensation condition, we design a photonic crystal heterostructure as schematically illustrated in Figure 5, whose spatial arrangement is represented as (HC)^N^D(HC)^M^. Within this structure, the H layer is a hyperbolic metamaterial (HMM) layer with a total thickness of *d_H_* = 132 nm; in practical implementation, this layer is equivalently represented by a subwavelength metal-dielectric alternating multilayer with a period number of 2 (denoted as (AB)^2^), which comprises alternating silver (Ag) layers (layer A) and titanium dioxide (TiO_2_) layers (layer B) with equal thickness, i.e., *d_A_* = *d_B_* = 33 nm. The C layer is a conventional dielectric TiO_2_ layer with a thickness of *d_C_* = 25 nm. Additionally, the D layer is a metallic Ag defect layer with a thickness of *d_d_* = 20 nm inserted at the center of the heterostructure. The variables N and M denote the period numbers of the one-dimensional photonic crystals on both sides of the defect layer, respectively.

**Figure 6:**
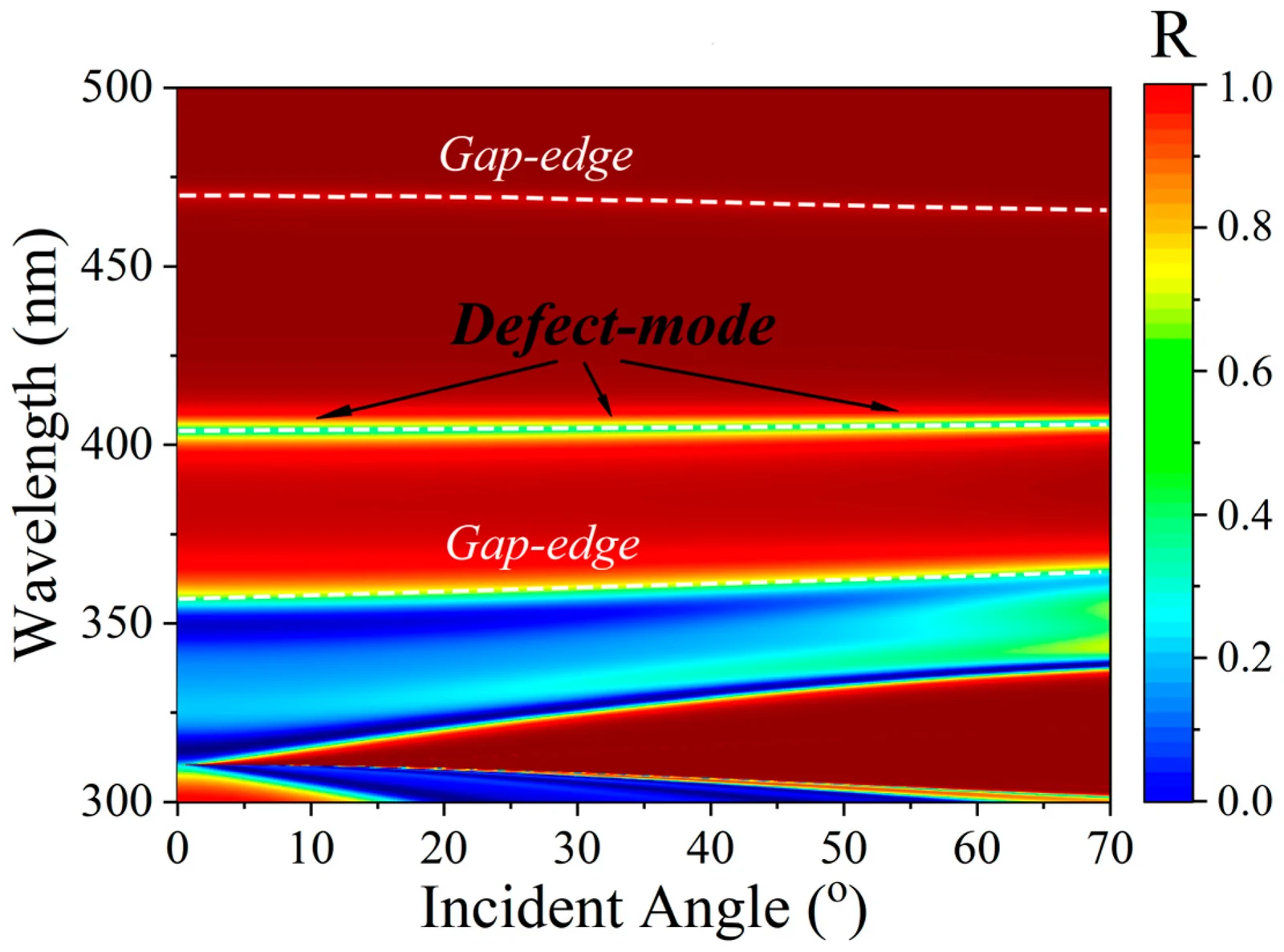
Simulated reflection spectra of the (HC)^3^D(HC)^4^ heterostructure as a function of the incident angle.

In this study, the period numbers N = 3 and M = 4 are selected to construct the (HC)^3^D(HC)^4^ photonic heterostructure, and its reflection spectra are numerically calculated using the transfer matrix method [26,27] shown in Figure 6. To circumvent the optical absorption interference induced by the imaginary part of silver’s permittivity and to clearly present the intrinsic evolution characteristics of the pure photonic bandgap, only the real part of the silver permittivity is incorporated into the simulations. The spectral results indicate that this structure forms a complete photonic bandgap in the wavelength range of 343.9 nm~453.6 nm. Further analysis of the spectral evolution under incident angles ranging from 0° to 70° reveals that the long-wavelength band edge remains almost unshifted, while the short-wavelength band edge merely undergoes a slight redshift. The combination of these two behaviors enables the structure to acquire an angle-insensitive omnidirectional photonic bandgap. Simultaneously, a localized defect mode is excited within the bandgap at a wavelength of 402.7 nm, manifesting as a distinct reflection dip in the spectra. As the incident angle increases from 0° to 70°, the resonant wavelength of this defect mode hardly shifts. This result shows that the proposed heterostructure achieves an angularly robust localized defect mode.

**Figure 7:**
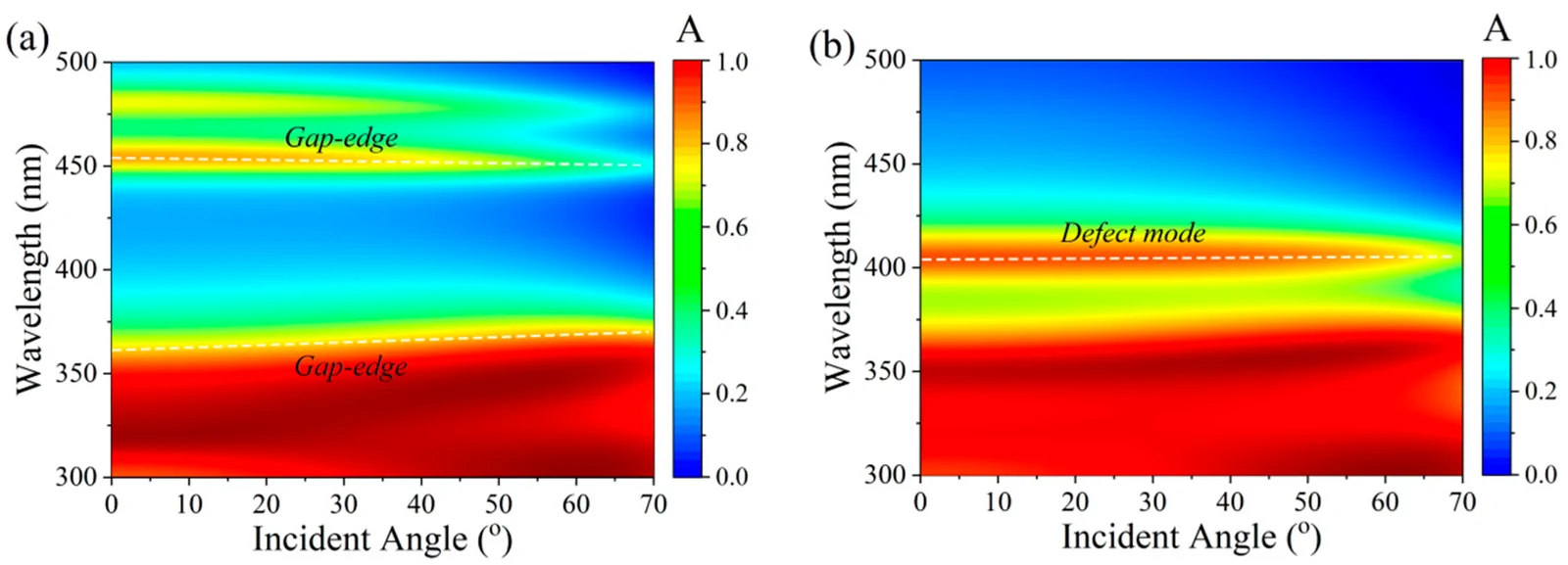
Calculated absorption spectra of (**a**) the photonic crystal (HC)^3^ and (**b**) the defect heterostructure (HC)^1^D(HC)^2^. A is the abbreviation for absorption.

Considering practical fabrication and the high ohmic loss of silver, too many metal layers will introduce strong background absorption, which completely masks the resonance of the defect mode. Therefore, we reduce the period numbers of the structure. The adjusted photonic heterostructure is denoted as (HC)^1^D(HC)^2^. Figure 7 shows the calculated absorption spectra of the basic structure and the defect heterostructure (HC)^1^D(HC)^2^. Under normal incidence (0°), the photonic crystal exhibits a complete photonic bandgap in the wavelength range of 342.1 nm–454.1 nm. After inserting the defect layer, a distinct absorption peak is excited inside the bandgap at the wavelength of 402.7 nm, which originates from the highly localized defect mode. More importantly, as the incident angle increases, the central wavelength of this absorption peak hardly shifts. This result indicates that the simplified structure not only overcomes the high absorption caused by multiple metal layers, but also successfully achieves an angularly robust localized defect mode.

**Figure 8:**
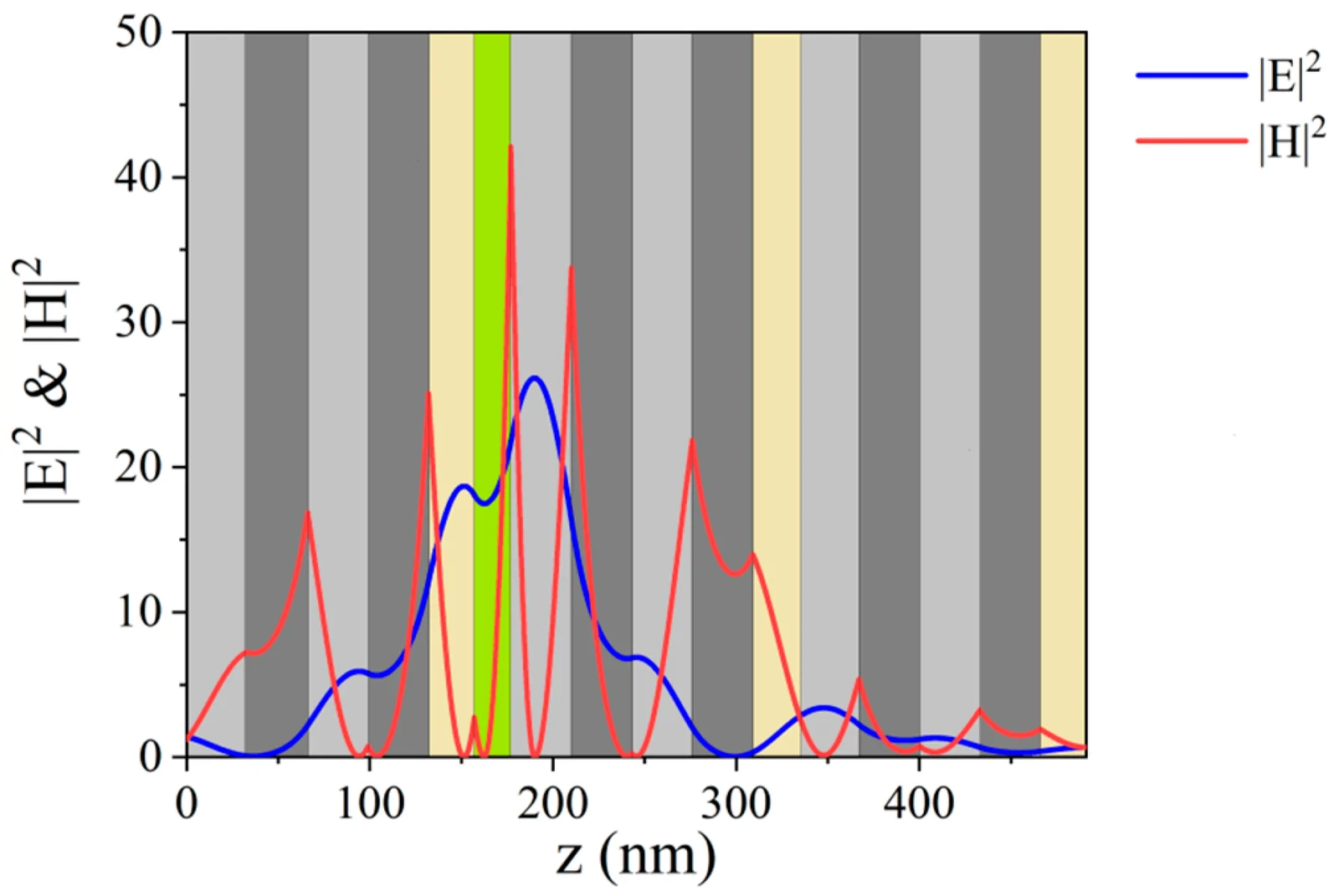
Electromagnetic field distribution of the (HC)^1^D(HC)^2^ photonic heterostructure at the resonant wavelength of 402.7 nm. The blue and red lines represent the electric field and magnetic field distributions, respectively.

To reveal the physical mechanism of this absorption peak, we calculate the electromagnetic field distribution of the (HC)^1^D(HC)^2^ photonic heterostructure at the resonant wavelength of 402.7 nm, as shown in Figure 8. The calculated results show that the electromagnetic energy is strongly localized within the central defect layer (layer D) and its adjacent interfaces. With the incident field amplitude set to unity, the local electric and magnetic fields inside the defect layer are enhanced by factors of 42.1 and 26.2, respectively. Such pronounced spatial confinement and field enhancement confirm the excitation of the localized defect mode in the heterostructure. As the incident angle increases from 0° to 70°, the resonance wavelength of the defect mode shifts by only 1.9 nm, corresponding to a relative shift of 0.47% with respect to the central wavelength of 402.7 nm. In contrast, the conventional all-dielectric defect-mode photonic crystal (Figure 1) exhibits a resonance wavelength shift of 62.0 nm over the same angular range, corresponding to a relative shift of 15.5% relative to 400.0 nm. This comparison demonstrates that the angular sensitivity of our design is reduced by approximately a factor of 33 compared with that of conventional all-dielectric structures.

## 3. Experimental Validation

Based on the designed parameters, we fabricated the (HC)^1^D(HC)^2^ sample on substrates using ion-assisted electron-beam evaporation under high vacuum. The structure was deposited on BK7 substrates using a Nanguang Machinery ZZS900-3/G box-type vacuum coating machine, Chengdu, China. For the Ag layers, the chamber was evacuated to 7.0 × 10^−4^ Pa, the substrate temperature was set at 150 °C, and the deposition rate was 46 nm/min. For the TiO_2_ layers, the chamber pressure was maintained at 2.0 × 10^−2^ Pa, with Ar + O_2_ ion-assisted deposition at an optimized oxygen partial pressure of 1.0 × 10^−2^ Pa; the gas flow rates of Ar and O_2_ were 6.0 SCCM and 7.0 SCCM, respectively, and the deposition rate was 30 nm/min.

**Figure 9:**
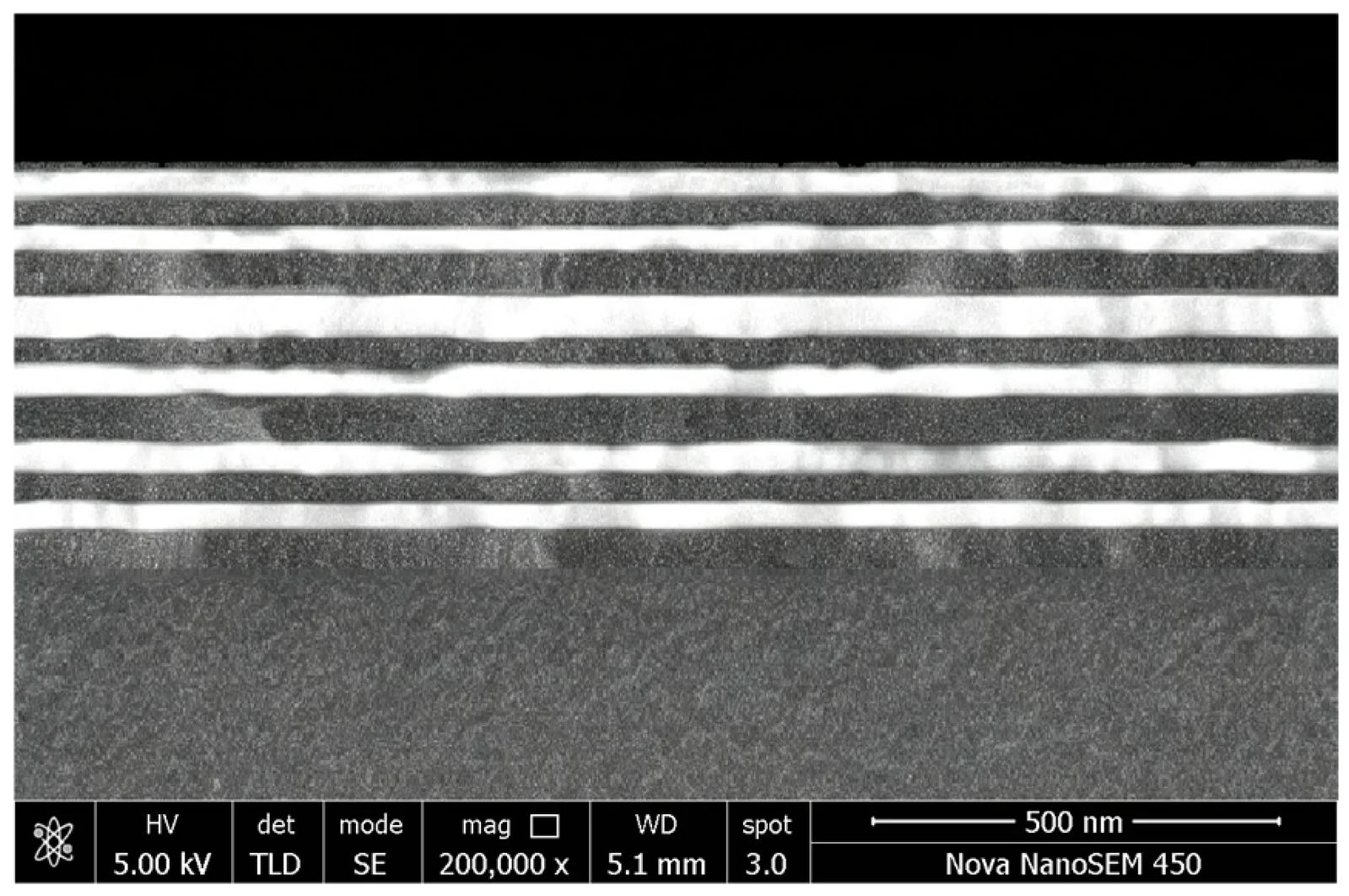
Cross-sectional SEM image of the fabricated (HC)^1^D(HC)^2^ heterostructure.

**Figure 10:**
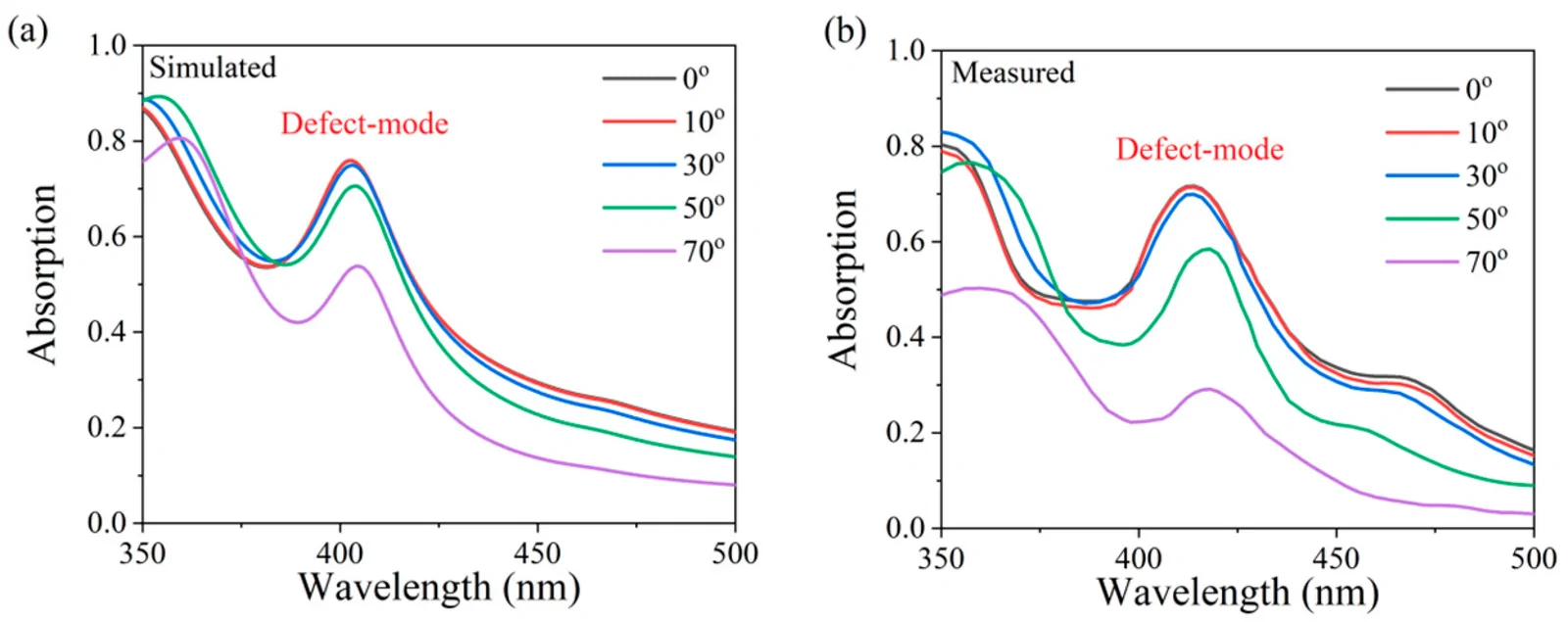
Calculated and measured absorption spectra of the photonic heterostructure: (**a**) simulated results and (**b**) measured results.

The cross-sectional morphology of the fabricated (HC)^1^D(HC)^2^ heterostructure was examined by scanning electron microscopy (SEM). As shown in Figure 9, the individual Ag/TiO_2_ bilayers within the HMM layers, the central Ag defect layer, and the adjacent dielectric layers are clearly resolved. The measured layer thicknesses are in good agreement with the design values, confirming the structural fidelity of the fabricated sample. Then, we measured the absorption spectra (A = 1-R-T) for TM polarization at five incident angles (0–70°) using a PerkinElmer LAMBDA 1050 spectrophotometer, Waltham, MA, USA. Figure 10 compares the calculated and measured absorption spectra of the photonic heterostructure. Figure 10a shows the simulated results, and Figure 10b shows the measured results.

The calculated results show that at incident angles of 0°, 10°, 30°, 50° and 70°, the resonant wavelengths of the defect mode are 402.7 nm, 402.8 nm, 403.2 nm, 403.8 nm, and 404.6 nm, with absorptances of 0.759, 0.757, 0.748, 0.708, and 0.540, respectively. As the incident angle increases from 0°to 70°, the resonant wavelength only shifts by 1.9 nm. Correspondingly, the measured absorption peak wavelengths at 0°, 10°, 30°, 50° and 70° are 414.3 nm, 414.4 nm, 417.8 nm, 413.5 nm, and 417.8 nm, with absorptances of 0.717, 0.715, 0.700, 0.584, and 0.292, respectively. In the same angle range, the maximum shift of the measured resonant wavelength is only 3.5 nm.

The measured results agree well with the calculated ones, verifying the angular robustness of the defect mode. The ~10 nm redshift mainly results from thickness errors during electron beam evaporation (typically ±3–5% per layer) and deviations in the actual refractive indices of TiO_2_ and Ag from the simulated values. Importantly, this offset is systematic and does not affect the angular response: the measured 0–70° drift is 3.5 nm, in good agreement with the simulated drift of 1.9 nm. In addition, the fabricated subwavelength silver films have extra non-radiative losses caused by surface roughness and grain boundary defects. Therefore, the actual loss is larger than the imaginary part of the silver permittivity used in the simulation, which leads to lower measured absorptances compared to the calculated results.

## 4. Discussion and Conclusions

While the proposed 1D planar heterostructure—composed of subwavelength Ag/TiO_2_ multilayers—significantly simplifies fabrication compared to complex higher-dimensional absorbers, the measured absorptance degrades at large incident angles. This degradation is primarily attributed to non-radiative losses amplified by surface roughness and grain boundary defects in the evaporated silver films. To approach the theoretical limit of near-unity absorption without compromising the angular robustness, future work can integrate alternative low-loss plasmonic materials. As shown in Table 1, conventional defect PCs [28,29] rely on fixed-dispersion materials, which limit the flexibility in tailoring their angular responses. Higher-dimensional PCs [30,31,32] can achieve angle-insensitive resonance wavelength optical properties, yet they usually require complex structures and fabrication processes. Previous HMM-based designs [23,24,25] realized omnidirectional or redshifted bandgaps but did not explore localized defect-mode absorption. In contrast, our proposed one-dimensional HMM-based heterostructure with a metallic defect layer achieves angle-insensitive defect-mode absorption, combining structural simplicity with excellent angular stability.

In conclusion, we have theoretically proposed and experimentally verified an optical absorber with angle-insensitive resonance wavelengths, based on the phase compensation effect of hyperbolic metamaterials. By inserting a metallic defect layer into the one-dimensional photonic crystal heterostructure, the inherent Bragg-induced blueshift is effectively overcome. Experimental measurements confirm excellent angular stability for TM polarization, with a wavelength shift of only 3.5 nm over the incident angle range of 0–70°. This work not only provides experimental insight into the phase control mechanism of metamaterials in nanophotonics but also offers a scalable route for developing wide-angle photodetectors, omnidirectional filters, and efficient thermal radiation devices.

## Figures and Tables

**Table 1:** Comparison of representative photonic crystal structures.

No.	Photonic Crystal Structure	Materials	Function	Angle-Sensitive?	Operating Band
28	1D defect PCs	with a chiral defect layer	Enhanced transmission	No	-
29	1D defect PCs	with a nanocomposite defect layer	Enhanced absorption	No	Infrared
30	3D PCs	3D-printed Ti-Nano resin	Complete band gap	Yes	Visible
31	3D PCs	porous anodic aluminum oxide	Complete band gap	Yes	Visible & IR
32	2D PCs	2D square array with high-dielectric	Directional reflection	Yes	Infrared
23	1D PCs	hyperbolic metamaterials	Omnidirectional band gap	Yes	UV–Vis
24	1D PCs	hyperbolic metamaterials	Optical absorbers	Yes	UV–Vis
25	1D PCs	hyperbolic metamaterials	Redshift band gaps	No (Redshift)	UV–Vis
**This work**	1D defect PCs	hyperbolic metamaterials	Optical absorbers	Yes	UV–Vis

## Data Availability

The raw data supporting the conclusions of this article will be made available by the authors on request.

## References

[1] Landy N.L., Sajuyigbe S., Mock J.J., Smith D.R., Padilla W.J. (2008). Perfect metamaterial absorber. Phys. Rev. Lett..

[2] Xie Z., Zhu X., Deng Y., Chen Y. (2023). A tunable wide-angle narrowband perfect absorber based on an optical cavity containing hyperbolic metamaterials. Phys. Chem. Chem. Phys..

[3] Yang G., Xiao Q., Zhang Z., Yu Z., Wang X., Lu Q. (2025). Exploring AI in metasurface structures with forward and inverse design. iScience.

[4] Hasan M.S., Islam M.T., Mansor M.F., Samsuzzaman M., Alsaif H., Soliman M.S. (2025). Spider-net shaped dual band angle insensitive metamaterial absorber for EMI shielding and energy harvesting application. Opt. Laser Technol..

[5] Pan J.R., Zhang H.F. (2025). Angle-insensitive temporal multi-channel narrowband absorber using discrete photonic time crystals. Appl. Phys. Lett..

[6] Yablonovitch E. (1987). Inhibited Spontaneous Emission in Solid-State Physics and Electronics. Phys. Rev. Lett..

[7] John S. (1987). Strong localization of photons in certain disordered dielectric superlattices. Phys. Rev. Lett..

[8] Kaliteevski M., Iorsh I., Brand S., Abram R.A., Chamberlain J.M., Kavokin A.V., Shelykh I.A. (2007). Tamm plasmon-polaritons: Possible electromagnetic states at the interface of a metal and a dielectric Bragg mirror. Phys. Rev. B.

[9] Liu K., Zheng C., Gao S., Chen X., Zhang S., Cao T. (2025). Optically reconfigurable Tamm plasmonic photonic crystals for visible spectrum. Engineering.

[10] Fink Y., Winn J., Chen C., Michel J., Joannopoulos J.D., Thomas E.L. (1998). A Dielectric Omnidirectional Reflector. Science.

[11] Wang X., Hu X., Li Y., Jia W., Xu C., Liu X., Zi J. (2002). Enlargement of omnidirectional total reflection frequency range in one-dimensional photonic crystals by using photonic heterostructures. Appl. Phys. Lett..

[12] Ferrari L., Wu C., Lepage D., Zhang X., Liu Z. (2015). Hyperbolic metamaterials and their applications. Prog. Quantum Electron..

[13] Poddubny A., Iorsh I., Belov P., Kivshar Y. (2013). Hyperbolic metamaterials. Nat. Photonics.

[14] Smith D.R., Schurig D. (2003). Electromagnetic wave propagation in media with indefinite permittivity and permeability tensors. Phys. Rev. Lett..

[15] Liu Z., Lee H., Xiong Y., Suun C., Zhang X. (2007). Far-field optical hyperlens magnifying sub-diffraction-limited objects. Science.

[16] Narimanov E.E. (2014). Photonic hypercrystals. Phys. Rev. X.

[17] Xue C., Ding Y., Jiang H., Li Y., Wang Z.S., Zhang Y.W., Chen H. (2016). Dispersionless gaps and cavity modes in photonic crystals containing hyperbolic metamaterials. Phys. Rev. B.

[18] Wu F., Lu G., Xue C., Jiang H., Guo Z., Zheng M., Chen C., Du G., Chen H. (2018). Experimental demonstration of angle-independent gaps in one-dimensional photonic crystals containing layered hyperbolic metamaterials and dielectrics at visible wavelengths. Appl. Phys. Lett..

[19] Guo Z., Jiang H., Chen H. (2020). Hyperbolic metamaterials: From dispersion manipulation to applications. J. Appl. Phys..

[20] Wei Q., Bi D., Ruan J., Zhou C., Wu F. (2026). Robust omnidirectional perfect absorption empowered by angle-immune topological interface states in one-dimensional photonic crystals heterostructures comprising hyperbolic metamaterials. Opt. Express.

[21] Shen S., Chew X.Y., Jing Q., Wu X., Dai J. (2025). Angle-independent coupling between topological interface states and Tamm states in photonic crystal heterostructures with hyperbolic metamaterials. Appl. Phys. Lett..

[22] Shen S., Hameed M.F.O., Zhang C., Zhu G., Qin F., Jiang M., Dai J. (2025). Tunable teraherz absorber via strong dual-Tamm plasmon coupling in graphene-MoS_2_ based photonic crystals. Phys. Lett. A.

[23] Lu G., Zhou X., Zhao Y., Zhang K., Zhou H., Li J., Diao C., Liu F., Wu A., Du G. (2021). Omnidirectional photonic band gaps in one-dimensional photonic crystals containing hyperbolic metamaterials. Opt. Express.

[24] Lu G., Wu F., Zheng M., Chen C. (2019). Perfect optical absorbers in a wide range of incidence by photonic heterostructures containing layered hyperbolic metamaterials. Opt. Express.

[25] Wu F., Lu G., Guo Z., Jiang H., Xue C., Zheng M., Chen C., Du G., Chen H. (2018). Redshift gaps in one-dimensional photonic crystals containing hyperbolic metamaterials. Phys. Rev. Appl..

[26] Yeh P. (1988). Optical Waves in Layered Media.

[27] Palik E. (1998). Handbook of Optical Constants of Solids.

[28] Lee K.J., Wu J.W., Kim K. (2015). Defect modes in a one-dimensional photonic crystal with a chiral defect layer. Opt. Mater. Express.

[29] Entezar S.R. (2021). Bistable absorption in a 1D photonic crystal with a nanocomposite defect layer. Appl. Opt..

[30] Zhang W., Yang J.K.W. (2024). 3D printed photonic crystals with a complete bandgap in the visible range. Nat. Nanotechnol..

[31] Roslyakov I.V., Kushnir S.E., Novikov V.B., Dotsenko A.A., Tsymbarenko D.M., Sapoletova N.A., Murzina T.V., Stolyarov V.S., Napolskii K.S. (2024). Three-Dimensional Photonic Crystals Based on Porous Anodic Aluminum Oxide. J. Phys. Chem. Lett..

[32] Zaremanesh M., Noori M. (2019). All-angle polarization-insensitive negative refraction in high-dielectric photonic crystal. Appl. Opt..

